# Challenging the prescientific frameworks of criminal justice: neurobiology and criminolytic interventions in the legalome era

**DOI:** 10.3389/fpsyg.2026.1791262

**Published:** 2026-04-16

**Authors:** Alan C. Logan, Pragya Mishra, Colleen M. Berryessa, Gregg D. Caruso, Fiona A. Hagenbeek, Thomas F. Denson, Jake M. Robinson, Susan L. Prescott

**Affiliations:** 1 Center for Justice and Mental Well-being, Nova Institute for Health, Baltimore, MD, United States; 2 Department of Law, University of Allahabad, Prayagraj, India; 3 School of Criminal Justice, Rutgers University, Newark, NJ, United States; 4 School of Business, Fairfield University, Fairfield, CT, United States; 5 Department of Biological Psychology, Vrije Universiteit Amsterdam, Amsterdam, Netherlands; 6 Amsterdam Public Health (APH) Research Institute, Amsterdam, Netherlands; 7 School of Psychology, University of New South Wales, Sydney, NSW, Australia; 8 College of Science and Engineering, Flinders University, Bedford Park, SA, Australia; 9 School of Medicine, University of Western Australia, Perth, WA, Australia; 10 Department of Family and Community Medicine, University of Maryland School of Medicine, Baltimore, MD, United States

**Keywords:** auto-brewery syndrome, behavioral epigenetics, biological criminology, hologenome, legalome, microbiome, neuroethics, rehabilitation

## Abstract

Contemporary systems of criminal justice are rooted in prescientific folk psychology assumptions related to moral fiber, free will, agency, and near-universal levels of willpower. For instance, people often believe that morally “defective” people make voluntary choices, failing to utilize their own self-control capacities. Given the wide acceptance of such beliefs, they are considered “normative” within law, serving to underpin retributive punishment. However, rapid advances in biological sciences—aided by multi-omics technologies—have illuminated the ways in which brain architecture and current metabolic conditions can constrain agency and shape here-and-now decision-making. Here in this perspective article, we examine how these advances are placing the prescientific foundations of criminal justice systems under duress. Older single-gene and single neuroimage attempts at explaining criminal behavior are giving way to the legalome era. This describes a more holistic epoch in which the simultaneous integration of mass biological data (e.g., polygenetic, metabolic, microbiome, metabolomic, lipidomic, and other omics-derived information) can provide explanatory power to criminal behavior and vulnerability, and guide personalized approaches to prevention and treatment. Advances in behavioral epigenetics are revealing how genetic predispositions interact with the exposome to shape human behavior through dynamic, potentially modifiable mechanisms. Moreover, evidence suggests that the human microbiome acts as a dynamic interface between environment and brain, influencing behavior in ways that are relevant to vulnerability, impulse control, and decision-making. Promising criminolytic interventions range from nutritional and pharmaceutical approaches to cognitive-behavioral therapy and contemplative practices. The integration of biological evidence and science education, along with ethically guided neurointerventions, will be critical to more humane systems of judgement.

## 1 Introduction

Rapid advances in neurobiology, behavioral genetics, and microbiome sciences—aided by multi-omics technologies—are fundamentally reshaping our understanding of human decision-making and behavior. Transdisciplinary evidence now indicates that cognition and action reflect current brain architecture shaped by accumulated exposures, including social and physical environments operating across developmental, historical, and even intergenerational timescales (Callender, 2021; Sapolsky, 2024). Older single-gene and single-neuroimage attempts to explain behavior are giving way to what we term the *legalome era*: a more holistic epoch in which simultaneous integration of mass biological data can provide explanatory power to criminal behavior and vulnerability, as well as guidance in personalized approaches to prevention and treatment. While some evidence directly challenges the coherence of free will itself (Sapolsky, 2023), the broader literature—from neuroscience (including neuromicrobiology and neuroendocrinology), behavioral genetics, exposome sciences, and the developmental origins of health and disease (DOHaD) construct—demonstrates substantial constraints on individual agency (Logan et al., 2025a).

These scientific advances stand in stark contrast to the foundations of contemporary criminal justice systems, which remain grounded in prescientific folk-psychological assumptions about free will, stable character, moral fiber, and near-universal willpower (Sapolsky, 2024; Logan and Prescott, 2025). In the United States, free will is commonly understood as a largely unconstrained capacity to make desire-driven choices (Lam and Yeung, 2025). These normative beliefs underpin retributive punishment, while limiting investments in prevention, treatment, and rehabilitation (Caruso, 2021; Logan et al., 2025b). Up to now, Western criminal justice systems have largely relegated neurobehavioral sciences (and the subject of neurodivergence) to the periphery, with minimal influence on determinations of blameworthiness, mitigation, prevention, or rehabilitation (Callender, 2021).

Courts continue to operate under the assumption of “*the responsible human subject as the noumenal self, characterized exclusively by a rational free will unencumbered by character, temperament, and circumstance”* (Dan-Cohen, 1992). This framework sustains a general disinterest in gene–environment interactions and other contextual determinants of behavior (Caruso, 2021). Stephen J. Morse, a prominent critic of biobehavioral evidence (Morse, 2018), has argued that “*it is simply not that hard to obey the law*,” and that suffering imposed through punishment is justified because offenders “*deserve it*” (Morse, 1984). Simultaneously, folk beliefs surrounding neurodivergence and complex brain conditions perpetuate notions of “evil” people and permanent dangerousness. Such beliefs are used to justify enhanced punishment (Greene and Cahill, 2012; Mowle et al., 2016; Allen et al., 2019; Berryessa, 2017; Silver et al., 2025; Jones and Cohen, 2025).

Drawing from the PsycINFO, CINAHL, Google Scholar, and PubMed databases, this perspective article examines how folk beliefs about free will are challenged by advances in neurobiology, neuroplasticity and the emerging legalome framework. We used a variety of search terms, including but not limited to, those related to neuroscience, violence, aggression, crime, behavior, neurodivergence, mental illness, free will, and words/terms associated with behavior genetics, microbiota and multi-omics (e.g., polygenetic, metabolic, microbiome, metabolomic, lipidomic) We also used Google Books and newspapers.com in efforts to identify and analyze relevant research (and more specifically, the historical deliberation of research). We argue that legalomic science reveals biology to be central to justice involvement without resorting to genetic or postcode determinism; it is acknowledged at the outset that genes cannot single-handedly determine complex human traits (Moore et al., 2025), and that an isolated environmental factor, such as residential zip/postal code, cannot independently determine behavioral health and related life course outcomes (Heckman and Eshaghnia, 2025).

We contend that a legalomic science offers a foundation for prevention-focused, rehabilitative approaches to justice, including the public health quarantine model. As described below, the public health quarantine model is a preventive and non-retributive approach that recognizes human behavior as emergent from complex biological, social, and environmental determinants. The legalome era emphasizes the protection of society without retributive punishment.

## 2 Folk psychology and resistance to biomedical criminology

Folk psychology refers to “commonsense” beliefs about human behavior, particularly assumptions concerning free will, agency, and moral responsibility. A familiar example—endorsed even by many health professionals—is the belief that body weight is controllable through willpower and personal agency (Burki, 2021). Extensive evidence from obesity science, especially neurobiology, directly contradicts this view (Rubino et al., 2020; Grannell et al., 2021). Similar gaps exist between scientific knowledge and folk beliefs about free will more broadly, including the persistent but erroneous belief that same-sex behavior can be altered through willpower alone (Logan et al., 2025a). At the close of the 20th century, a majority of Americans (56%) endorsed this view (Leland and Miller, 1998), and such beliefs remain associated with discriminatory judgments (Sharifi and Trau, 2025).

Obesity, sexual orientation, and neurodivergence illustrate how agency beliefs held by one person are tightly linked to the moral judgment of others. Experimental studies show that when hypothetical offenders are described as obese or homosexual, guilt is more readily assumed and punishments are harsher (Schvey et al., 2013; White II et al., 2014; Wiley and Bottoms, 2009; Mirabito and Lecci, 2021). Both groups are overrepresented in criminal justice systems in the Global North (Meyer et al., 2017; Mosomi et al., 2025). Stronger belief in unconstrained free will consistently predicts greater blame attribution—for obesity and mental illness (Chandrashekar, 2020), harsher criminal punishment (Walton and Bastian, 2025), victim blaming (Genschow and Vehlow, 2021), and endorsement of “suffering” and “getting even” as an inherent component of punishment (Snater, 2019). These beliefs also support broader moral worldviews in which outcomes are seen as reflections of moral deservingness (Carey and Paulhus, 2013).

Folk psychological beliefs frequently conflict with contemporary science. Despite overwhelming evidence that addiction is a neurobiological disease, individuals with strong agency beliefs are more likely to frame addiction as a moral failing and support punitive responses (Rise and Halkjelsvik, 2019; Kennedy-Hendricks et al., 2017). Similarly, despite robust evidence documenting the psychological harms of solitary confinement (Giguère et al., 2025), corrections personnel with stronger agency beliefs are less likely to acknowledge these harms (Aranda-Hughes and Mears, 2025). Strong free will beliefs are also associated with inflated perceptions of one’s own objectivity and resistance to bias (Chandrashekar et al., 2021). In contrast, education in biological sciences and exposure to neuroscientific explanations of behavior are linked to weaker free will beliefs and reduced support for retributive punishment (Shariff et al., 2014; Thomaidou and Berryessa, 2022).

Educational context is therefore central. Criminal justice degree programs remain dominated by social theories and exhibit sustained resistance to biological perspectives on behavior (Wright et al., 2008; Beaver, 2019). In a 2008 review of U.S. criminal justice PhD programs, fewer than 2% of faculty expressed interest in biological explanations of crime, and formal training in biological sciences was virtually absent (Wright et al., 2008). Lawyers and judges likewise tend to have limited scientific training, including in biology and neuropsychology (Gatowski et al., 2001; Costanzo and Krauss, 2021), while often overestimating their scientific competence—a manifestation of the Dunning–Kruger effect (Lorandos and Blevins, 2025).

Resistance to biology also persists within sociology, where some scholars continue to argue that biological theories of crime have “failed to withstand the test of time” (Rollins et al., 2025). Yet convergent evidence from neuroscience (Sapolsky, 2024), nutritional neuroscience (Prescott et al., 2024b), microbiology (Logan et al., 2025c), toxicology (Shaffer et al., 2025; Yıldız et al., 2023), infectious disease (Bransfield, 2025), brain architecture (Pieperhoff et al., 2025; Miller et al., 2024), traumatic brain injury (Theadom et al., 2023; Ineson et al., 2023), neurodivergence (Wilson et al., 2024), endocrine markers (Armstrong et al., 2022; Rzepczyk et al., 2024), and cardiovascular markers (Ling et al., 2026), consistently links biological factors to justice involvement and decision-making. At the population level, the overwhelming male dominance of global cross-cultural, prison populations (≈95%) provides a Holmesian clue that biology matters (Clegg et al., 2024).

In the planetary health context, multiple studies show that acute heat events and airborne particulate matter exposure are associated with time-locked increases in violence and criminal behavior (Awaworyi Churchill et al., 2023; Choi et al., 2024; Berman et al., 2019; Cuartas and Camacho, 2025)—findings that are likely mediated by biological mechanisms. The administration of dopamine agonist drugs (e.g., the restless leg syndrome drug ropinirole) have been consistently linked to risk taking, addictive behaviors, dysfunction of impulse control, and justice involvement (Moore et al., 2014; Wolfschlag and Håkansson, 2021; Titheradge, 2026). The overall theory that biology matters to justice involvement is not only durable but supported by volumes of research (Sapolsky, 2023).

At the same time, sociology provides an essential corrective by foregrounding structural injustice. Across jurisdictions, Black and Indigenous peoples, along with other ethnoracial minorities, migrants, and foreign nationals, experience disproportionate justice involvement (Clegg et al., 2024). Skepticism toward biological explanations is historically grounded, given early 20th-century abuses rooted in racist theories of criminality that otherwise contributed to eugenics, compulsory sterilization, and coercive “rehabilitation” practices (Arford and Madfis, 2022; King et al., 2022).

By the 1970s, biomedical criminology had been widely dismissed as pseudoscientific and harmful (Nassi and Abramowitz, 1976). Through the 1980s and 1990s, biological and individual-difference explanations ranked near the bottom of perceived causes of crime within criminology (Ellis and Hoffman, 1990; Ellis and Walsh, 1999). Notably, this period coincided with the rise of mass incarceration, expanding prosecutorial power, rigid parole regimes, and stupendous increases in life-without-parole sentencing in the United States (Pfaff, 2017; Deitch, 2020; Wiggins et al., 2022).

The damage incurred by marginalizing biomedical criminology is difficult to quantify but likely profound (Schwartz et al., 2019). The criminalization of mental illness, neurodivergence, and complex brain disorders intensified, while emerging areas such as nutritional criminology were stifled (Logan and Schoenthaler, 2023). With neurobiology and biophysiology deemed irrelevant to criminal choice, folk narratives of agency and moral fiber prevailed, reinforcing punitive ideologies and moralized constructions of justice-involved individuals as “sinners” (Zeki et al., 2004).

In summary, debates about blameworthiness and rehabilitation unfold within a cultural landscape shaped by deeply entrenched assumptions about agency. Although calls to integrate biomedical perspectives into criminal justice are increasing (Callender, 2021; Das et al., 2026), comparatively little attention has been paid to the belief systems that sustain retributive frameworks (Caruso, 2021; Logan et al., 2025b). The United States remains an outlier in punitiveness (Clegg et al., 2024). Understanding how legal institutions and academic traditions continue to marginalize biological sciences is therefore central to reimagining justice (Logan and Prescott, 2025) and sets the stage for the discussion of behavioral plasticity and neuronormalization that follows.

## 3 Behavioral neuroplasticity and epigenetic evidence

Neuroplasticity refers to the nervous system’s capacity to modify neural pathways, synaptic strength, and functional organization in response to intrinsic and extrinsic stimuli across the life course. This adaptive capacity is supported by synaptic and structural plasticity, neurogenesis, and large-scale functional reorganization, enabling the brain to respond dynamically to changing internal states and environmental conditions (Gazerani, 2025). In short, neuroplasticity reflects ongoing reorganization with meaningful consequences for brain structure and function.

Although much neuroplasticity research emphasizes beneficial outcomes—such as learning, skill acquisition, and rehabilitation—plasticity is not inherently adaptive. Reorganization driven by chronic stressors or adverse exposures can entrench maladaptive patterns, helping to explain the persistence of neuropsychiatric pathology and behavioral dysregulation (Gazerani, 2025; Ren et al., 2025). Collectively, neuroplasticity research underscores that the brain is neither fixed nor irreversibly shaped in early life. While the developmental origins of health and disease (DOHaD) framework demonstrates the enduring influence of early-life conditions and genetics on later outcomes, evidence from adaptive plasticity shows that functional improvements in behavior and cognition remain possible across the lifespan.

Within this context, behavioral epigenetics examines how lived experiences and environmental exposures influence gene expression without altering DNA sequence. Through mechanisms including DNA methylation, histone modification, chromatin remodeling, and RNA-based processes, epigenetics provides a biological bridge between genotype and phenotype (Ng et al., 2023). As a core component of the DOHaD framework, behavioral epigenetics enables investigation of how accumulated experiences—particularly during sensitive developmental periods—shape long-term health and behavior (Hein et al., 2024).

Preclinical models further demonstrate that experimentally induced epigenetic modifications can alter behavior. For example, inhibition of histone deacetylase activity has been associated with reduced aggression and stress-related behaviors (Covington et al., 2009; Hernandez Carballo et al., 2024). Psychotropic medications and emerging “epidrugs,” including gut-microbe-derived histone deacetylase inhibitors, represent a growing frontier in neuropsychiatric research (Gladkova et al., 2023; Paeger, 2024; Paciolla et al., 2025). These developments extend epigenetics into the domains of neuronormalization and criminolytic intervention (Gaba et al., 2025).

In criminal justice contexts, behavioral epigenetics focuses on interactions among environmental exposures (e.g., adverse or positive childhood experiences), genetic vulnerability, and gene expression related to aggression, violence, and antisocial behavior (Refolo et al., 2025; Bohare, 2024). Exposure to violent social environments, for example, can become biologically embedded through DNA methylation processes (Berg et al., 2022; Leshem and Weisburd, 2019). On the victimology side, epigenetics helps explain individualized and long-term health consequences of violence and trauma (Bailo et al., 2024; Gemmati et al., 2025), including the well-documented victim–offender overlap (Alua et al., 2024; Berg and Schreck, 2022). Importantly, this field also highlights resilience: positive childhood experiences are associated with improved mental health, lower delinquency risk, stronger self-control, and healthier adult behaviors (Bethell et al., 2019; Sege et al., 2025; Sousa et al., 2025).

Genetic factors contribute substantially to behavioral variation, with estimates suggesting that 40–60% of variance in impulsive, aggressive, antisocial, and addictive behaviors is heritable (Ferguson, 2010; Rhee and Waldman, 2011; Bezdjian et al., 2011; Tuvblad and Baker, 2011). Twin studies, however, reveal the epigenome’s sensitivity to environmental influence. Monozygotic twins show epigenetic divergence from birth that increases over time as unique environmental exposures accumulate (Fraga et al., 2005; Talens et al., 2012; van Dongen et al., 2016). Such designs permit causal inference, demonstrating, for example, reversible methylation differences attributable to smoking rather than genetic liability (Tan, 2020; van Dongen et al., 2023). These epigenetic patterns track both exposure-related and disease-related processes, contributing to phenotypic divergence among genetically identical individuals (Kikuchi et al., 2021; Saffery and Bell, 2022; Drouard et al., 2024).

Epigenetic correlates of externalizing behaviors have been identified across candidate-gene and epigenome-wide studies, including associations with ADHD, conduct disorder, substance use, impulsivity, risk-taking, sexual offending, and aggression (Barker et al., 2018; Cecil and Nigg, 2022; Chiocchetti et al., 2022; Pishva et al., 2023; Mao et al., 2024; Androvičová et al., 2025; Meijer et al., 2023). Composite measures, such as accelerated epigenetic aging, are increasingly informative and have been linked to antisocial behavior, low self-control, suicide attempts, adverse life events, incarceration, early-life adversity, and the absence of positive social experiences (Berg et al., 2022; Langevin et al., 2022; Jokinen et al., 2023; Willems et al., 2024; Hogan et al., 2025; Prigerson et al., 2025). Meta-analyses confirm these associations across social determinants of health (Willems et al., 2025).

Together, these findings support a DOHaD-informed view of molecular continuity linking early-life conditions to adult inflammation, neuropsychiatric health, and biological aging (Raffington et al., 2024). Epigenetic signatures may thus serve as markers of “metabolic memory,” reflecting enduring cellular imprints of prior environmental conditions (Dong et al., 2024). Nonetheless, further longitudinal research is needed to disentangle confounding lifestyle factors (Ware et al., 2025).

Of course, behavioral epigenetics operates within broader efforts to understand genetic contributions to behavioral variation (Plomin, 2023). Early single-gene approaches—such as work on monoamine oxidase A variants linked to impulsive aggression—generated interest but limited explanatory power in legal contexts (Mentis et al., 2021; Farahany and Robinson, 2021). Contemporary research has shifted toward polygenic frameworks, identifying loci associated with risk-taking, mood instability, and antisocial behavior (Karlsson Linnér et al., 2019; Ward et al., 2020; Tielbeek et al., 2017), and links to risks of justice involvement (Tielbeek et al., 2022). Polygenic indices aggregate small effects across millions of loci and show meaningful associations with personality, attention, rule-breaking, and childhood externalizing behavior (Tanksley et al., 2025; Alajääskö et al., 2025; Schwaba et al., 2026). In isolation the indices are not suitable for routine clinical use. However, their predictive capacity is advancing (Andreassen et al., 2023; Wang et al., 2023) and will likely provide value when combined with other sources of bioinformatics.

Crucially, this body of work does not support genetic determinism. Polygenic indices are probabilistic summaries whose expression is shaped by environmental and social contexts (Alajääskö et al., 2025). Integrating genetic variation with epigenetic, microbiome, and multi-omics data offers a more nuanced understanding of risk and resilience (Czamara et al., 2021), a direction explored in the following section.

## 4 Legalomics—the new frontier of objectivity

The *legalome* describes the biological ecology of justice (Prescott et al., 2025). It integrates evidence across tightly linked domains—including the exposome (cumulative social and physical exposures), human multi-omics (e.g., genomic, epigenomic, transcriptomic, and metabolomic data), and the microbiome (including the hologenome)—to examine how reciprocal biological–environmental interactions shape behavior, health, performance, and culpability within criminal justice systems.

Metabolomics research increasingly demonstrates that small molecules, including those derived from gut microbe–mediated fermentation, influence energy metabolism, neurotransmission, oxidative stress, and behaviors relevant to justice involvement (Hagenbeek et al., 2020; Chen et al., 2020; Wang et al., 2021). Identification of distinct metabolic signatures and clustered metabolite profiles offers promise for improved diagnostics and targeted interventions (Salmerón et al., 2025). Advances in machine learning are now enabling the alignment of peripheral biomarkers—such as blood-based DNA methylation patterns—with brain physiology, moving multi-omics toward clinically relevant integration (Kullmann et al., 2025). When combined with polygenic indices, multi-omics approaches provide unprecedented resolution for understanding behavioral risk and informing personalized care, a potential further amplified by incorporating microbiome data.

Evidence now clearly demonstrates that gut microbes play a substantial role in cognition and behavior. The mechanistic pathways—described in detail elsewhere (Devason et al., 2024; García-Cabrerizo and Cryan, 2024)—include direct neural signaling (e.g., vagal and spinal afferents), as well as indirect immune, metabolic, and endocrine routes. Gut microbes also influence brain structure and function by shaping nutritional status through the synthesis, metabolism, and absorption of vitamins, minerals, fats, and polyphenols (Ephraim et al., 2022; Cassotta et al., 2025; Taherkhani et al., 2025). The combined genetic landscape of host and microbes—the hologenome—contributes to neurodevelopment and neuropsychiatric vulnerability, in part through microbiome-mediated effects on DNA methylation and gene expression (Dooling and Costa-Mattioli, 2025; Rubas et al., 2025; Gustafson et al., 2025).

Emerging evidence from the microbiome–gut–brain axis further complicates folk-psychological assumptions about unconstrained agency. Gut microbial communities and their metabolites can modulate stress responsivity, inflammatory tone and neuroactive signaling pathways that influence affect, threat processing and behavioral control (Bautista et al., 2025; Ramadan et al., 2025). While human causal evidence is still developing, convergent findings from mechanistic reviews and translational studies support the view that “here-and-now” decision-making is partly shaped by dynamic biological state – including microbially mediated immune and metabolic signaling – rather than willpower alone (Uzan-Yulzari et al., 2024; Langmajerová et al., 2025). For example, emergent research using structural magnetic resonance imaging (sMRI) demonstrates that gut microbiota and multi-omics data have strong predictive capabilities for both brain structural variations and the severity of behavioral symptoms in children with autism spectrum disorder (Mi et al., 2026).

This is important because “willpower” framing is strained by evidence that behavioral control can vary with *physiological* context—including immune and metabolic state and, potentially, gut microbial metabolism. This does not excuse harmful actions; rather, it strengthens the empirical case that behavioral capacity is not constant across people or time, and that the prevention and rehabilitation models (discussed below) should take seriously modifiable biological constraints (for example, stress-reactivity, inflammation, and metabolically driven shifts in affect and impulse control) (Zhao et al., 2025; Iktilat et al., 2026; Molina-Torres et al., 2019; Diotaiuti et al., 2025) that might otherwise bias judgement and risk-taking.

While legalomics has clear relevance for forensic neuropsychiatry and correctional health, its scope extends to professionals operating within justice systems themselves (Logan et al., 2025b). A legalome perspective offers insight into biological contributors to workplace performance, decision-making under stress, excessive use of force, fitness for duty, burnout, and organizational culture (Prescott et al., 2025). Substantial evidence shows that police officers, corrections personnel, judges, and lawyers—particularly public defenders working within punitive systems—experience high levels of chronic stress (Baćak et al., 2024). Yet the multi-layered biological ecology of justice work, and its influence on real-time decision-making, remains largely unexamined (Logan et al., 2025d).

## 5 Criminolytic interventions, neurotherapeutics

Evidence emerging from legalomics and associated informatics extends beyond theory, offering practical implications for personalized medicine and targeted criminolytic intervention. A wide range of pharmaceutical (da Cunha-Bang and Knudsen, 2021; Felthous et al., 2021; Palmieri and Clark, 2025), neuromodulatory (Sergiou et al., 2022; Camara et al., 2025), nutritional (Raine and Brodrick, 2024; Prescott et al., 2024b; Konkolÿ Thege et al., 2025), cognitive-behavioral (McCloskey et al., 2022; Morris et al., 2024), contemplative (O'Dean et al., 2025; Pankovics and Martinovic, 2024), and movement-based (Georgiadis et al., 2025) interventions have demonstrated potential to reduce aggression, impulsivity, antisocial behavior, and substance use among justice-involved populations. Legalomics provides a framework for matching individuals to interventions with greater specificity, timing, and relevance.

These approaches can be considered *neurotherapeutic* and/or *neurorehabilitative*. Since pathways to justice involvement often reflect differences in neurocognitive functioning, safe and effective interventions aimed at supporting brain function toward patterns observed in non-criminal populations are both ethically defensible and rehabilitative (Denson, 2021; Denson and Choy, 2024; Denson et al., 2024; Denson et al., 2025). Such interventions, which do not seek to “cure” neurodiversity, form a core component of the public health quarantine model of justice (Caruso, 2021).

The public health quarantine model argues that converging evidence from neurobiology and the social determinants of health undermines traditional justifications for retributive punishment. While society retains the right to protection, any deprivation of liberty should follow public health principles—particularly the principle of least infringement—where restraint is proportionate to demonstrated risk rather than moral blame or deterrence (Caruso, 2016). This model prioritizes prevention, treatment, and rehabilitation, and rejects suffering as a legitimate aim of punishment. Even when long-term incapacitation is required, the model provides no justification for misery beyond that necessary to mitigate harm (Caruso, 2021; Caruso, 2023).

The public health quarantine model acknowledges harms done to victims and recognizes the restorative justice approach as a bridge to implementation. Restorative approaches promote respectful, inclusive, and humane dialogue between the harmed and the justice-involved party, emphasizing healing and accountability in a way that gives victims and communities a meaningful voice while avoiding the cruelty of retribution. Research indicates that restorative justice can reduce recidivism while providing psychological benefits for both harmed and harming parties (Rossner and Taylor, 2024; Yeong and Moore, 2020; Bouffard et al., 2017).

Although neuronormalization is sometimes conflated with “moral bioenhancement,” the latter framing is incompatible with the quarantine model. The goal is not moral correction *per se*. Rather, it is to support neurocognitive functioning within existing constraints on agency—constraints well-established by transdisciplinary biological evidence (Sapolsky, 2023; Caruso, 2023). Nutritional agents such as omega-3 fatty acids, multivitamins, and probiotics, for example, are not “morality pills,” but may influence brain function in ways that reduce impulsivity, improve affect regulation, and enhance future-oriented decision-making (Chang et al., 2016; Dighriri et al., 2022; Ma et al., 2025; Zandifar et al., 2025; Shen et al., 2025; Musculus et al., 2025). Healthy dietary patterns may support neuropsychological functioning in vulnerable youth, reducing the risk of justice involvement (Gibbs and Beaver, 2026). These generally safe interventions may facilitate engagement with rehabilitative processes, much as educational programming, which is hardly controversial, supports pro-social skills development.

Some criminolytic lifestyle and nutritional interventions may also operate partly through microbiome-mediated mechanisms (for example, shifting microbial metabolites that influence immune and neuroendocrine signaling) (Prescott et al., 2024b). This possibility does not require microbiome determinism; it simply expands the intervention logic from a brain-only model to a systems model in which diet, sleep, stress, and social environment can alter biology – including gut microbial function – in ways that may change behavioral bandwidth and treatment responsiveness (Dinan and Cryan, 2017; Carbia et al., 2023; Neroni et al., 2021; Tonnelé et al., 2025). To date, siloed analyses of neuroimaging data have neglected the dynamic interplay between the brain and peripheral biological system (e.g., metabolic and microbiological ecosystems) (Mi et al., 2026). Integrating data could transform care and lead to a new vantage point—a holistic and personalized view of prevention, treatment and rehabilitation that might be seen as forensic neuroecology.

As mentioned, criminolytic interventions are constrained by existing brain architecture. Overestimation of efficacy—especially when driven by strong agency assumptions—risks reproducing blame when individuals fail to respond (Gordon et al., 2025). This pattern mirrors historical failures in obesity treatment, where non-responsiveness has been frequently attributed to lack of effort rather than biological constraint (Logan and Prescott, 2025). Similar risks arise in criminal justice when individuals are labeled “resistant” to treatment without adequate attention to neurobiological context.

Psychotherapy illustrates these limits. Contrary to common assumptions, evidence supporting its broad effectiveness in reducing recidivism among justice-involved populations is weak (Beaudry et al., 2021; Rosenfeld et al., 2024). Undoubtedly, some individuals will respond very well to psychotherapy. The question is, who is best suited, at a particular time, and why? Therapeutic impacts may be minimal when underlying biological factors—such as ultra-processed diets common in carceral settings (Prescott et al., 2024a), heavy metal exposure (Beckwith et al., 2021; Shaffer et al., 2025), omega-3 deficiency (Meyer et al., 2015), metabolic dysfunction, or microbiome-related conditions—remain unaddressed. It would be, as some have intimated, like placing a new chain on a bicycle and expecting it to function while still having two flat tires (Logan and Schoenthaler, 2023).

Similarly, contemplative practices are not universally benign; adverse effects, particularly among trauma-exposed individuals, are increasingly documented (Britton et al., 2021; Canby et al., 2025). Contemplative interventions warrant particular scrutiny within this framework. The rapid proliferation of secular mindfulness programs in Western correctional and therapeutic settings—often characterized as “McMindfulness”—can reproduce the very folk psychology assumptions the legalome perspective challenges. Standardized protocols assume uniform responsivity, frame non-response as insufficient commitment, and position the provider as expert while the subject is expected to respond positively. This mirrors the broad expectations revolving around the idea that agency-based behavioral/lifestyle interventions (otherwise ignoring neurobiological variability) should work for most, if not all, people living with obesity (Logan and Prescott, 2025; Goldkorn et al., 2025).

Traditional contemplative epistemologies—such as those underlying Vipassana practice (Goenka, 1987)—explicitly reject these assumptions, emphasizing individual variability in response, and non-linear trajectories of change (Buddhaghosa, 2020). There is recognition that transformation cannot be willed into existence, but emerges through interaction with existing biological and psychological conditions (Goenka, 1987). When approached with appropriate humility about neurobiological constraint and individual difference, contemplative practices show promise as criminolytic interventions with measurable biological endpoints (Mishra, 2024). For example, such interventions have been associated with shifts in DNA methylation patterns, inflammatory markers, HPA axis regulation, and gut-brain signaling (Kaliman et al., 2022; Kripalani et al., 2022; Sullivan et al., 2024; Carvalho Silva et al., 2024).

These observations underscore the relevance of whole-person health and exposome-informed personalized care. Individual placement along the vitality–disease continuum reflects interacting biological, environmental, behavioral, and social factors (Langevin, 2024). Across healthcare, one-size-fits-all interventions frequently underperform or worsen outcomes (Harbeck and Wuerstlein, 2019; Sugandh et al., 2023; Xie et al., 2025). Advances in exposomics and ecological personalized medicine now enable dynamic assessment of cumulative exposures and biological responses, supporting more precise and adaptive intervention strategies (Sarigiannis et al., 2025; Tan et al., 2025), including those responsive to neurodiversity and brain architecture (Mohammad et al., 2025).

Within corrections, the risk–need–responsivity model has long dominated practice. While appealing in theory, it remains heavily psychotherapy-centered and lacks strong empirical support (Andrews and Bonta, 2010; Fazel et al., 2024). For now, siloed neuroimaging provides limited value in predicting risk, and experts in the field suggest that functional brain network data will likely enhance the value of risk assessments and personalized care approaches (van Dongen et al., 2025). We agree with this contention. Emergent preprint evidence reveals that brain networks strongly overlap with the expression of genes previously associated with aggression and related clinical phenotypes (e.g., irritability, conduct disorder, antisocial personality disorder) (Dugré et al., 2025a). Structural network mapping, rather than the historical neuroscience focus on isolated brain regions, provides a unifying explanation for decades of inconsistent results in aggression and antisocial behavior (Dugré et al., 2025b).

We would add that incorporating legalomic markers may improve risk assessment and intervention matching, both in custodial settings and community corrections (Logan et al., 2025b; Logan et al., 2025e). For example, microbiome and multi-omics data are emerging as valuable to the identification of subthreshold mental disorders (Xian et al., 2026). Although exercise is often stated to be of value as a treatment modality for neuropsychiatric disorders, some people seem more responsive. Preliminary research involving adults with substance use disorder in a carceral setting indicates that baseline microbiome profiles can predict exercise treatment response with 91% accuracy; moreover path analysis indicated that approximately 70% of exercise effects on mood and psychological distress in the study population were mediated through the gut microbiota-short-chain fatty acid metabolite-neuroendocrine pathway (Yang et al., 2026).

Finally, the therapeutic lens of legalomics extends to justice institutions and personnel. Organizational conditions can become biologically embedded and propagate through systems via stress contagion. Metabolic stress, for example, has been linked to harsher judgments and punitive decision-making among justice professionals (Logan et al., 2025d). Burnout—often driven by leadership and organizational culture—is associated with impaired performance and excessive use of force, particularly in policing (Kop and Euwema, 2001; Queirós et al., 2013; Dyrbye et al., 2020; Burns et al., 2021; Gutschmidt and Vera, 2022), as well as metabolic dysregulation (Fernandez-Montero et al., 2019; Lennartsson et al., 2025). Despite growing attention to organizational wellness, few interventions examine biological endpoints (Wirth et al., 2014; Lockie et al., 2022; Bes et al., 2023). Indeed, it has recently been argued that in high-stakes professions, such as law enforcement, that it may be unethical to completely ignore biological factors (in pre-employment screening or fitness for duty assessments) that might otherwise predispose individuals to behaviors that could result in societal harm (Oprinca-Muja et al., 2026); at the very least biological markers could complement the “paper-and-pencil” tests and clinical opinions that are commonly used in screening of law enforcement candidates and personnel.

## 6 Ethical considerations

Retrospective critiques of harmful biological theories in criminal justice often overlook their historical context. Many such practices emerged during periods when medicine and public health operated largely without rigorous ethical frameworks or controlled research designs (Doll, 1998; Elzein, 2024). The rapid postwar expansion of crude psychosurgeries, including lobotomies—often performed on socially privileged patients—was facilitated precisely by the absence of ethical oversight and evidentiary standards (Valenstein, 1986).

Contemporary medicine and healthcare, by contrast, is governed by extensive ethical frameworks, particularly for research involving vulnerable populations (Buthut et al., 2025; Atkinson-Clement et al., 2025; Herrera-Ferrá et al., 2025). Justice-involved and incarcerated individuals fall squarely within this category and warrant heightened protections. However, the legacy of early 20th-century abuses has produced policies that, in some jurisdictions, exclude incarcerated people entirely from consenting to or participating in ethically informed research (Ahalt et al., 2018; Wurcel et al., 2023; Gaber et al., 2025). As a result, those most likely to benefit from advances in health, wellbeing, and biologically informed justice—while also challenging entrenched folk psychology assumptions—are often denied participation and voice.

To navigate the ethical and practical challenges of integrating biological insights into law, we propose four guiding principles:

1. *Contextualized interpretation and graduated responsibility*: Biological data should be interpreted alongside environmental, psychological, and cultural factors, moving beyond binary notions of culpability toward models that recognize both agentic capacity and neurobiological constraint. Some countries, such as the Netherlands, avoid a dichotomy between the non-medical/scientific terms of “sane” versus “insane” (wherein almost everyone is considered “sane”) and instead base judgements around responsibility, diminished responsibility, and complete insanity.
2. *Rehabilitation-focused application*: Legalomic insights should prioritize prevention, rehabilitation, and support, rather than serve as mechanistic or deterministic “biological alibis.” Within a public health quarantine model, emphasis is placed on neuroplasticity and lifelong epigenetic responsiveness.
3. *Cultural humility and cross-validation*: Findings derived largely from Western populations require validation across diverse cultural, social, and genetic contexts prior to legal application.
4. *Procedural safeguards*: Clear standards for admissibility, proportionality, and scientific validity—including peer review, replication, and post-implementation monitoring—are essential.

These principles are not definitive solutions, but starting points for dialogue among scientists, legal scholars, ethicists, and cultural practitioners. Specific ways to operationalize the legalome in systems of criminal justice have been described in detail elsewhere (Mishra et al., 2026). These include standardized clinical screening and referral with role clarity, the use of standardized protocols via accredited laboratories, expert testimony that distinguishes between bounded, testable metabolite–impaired pathways (e.g., endogenous ethanol in auto-brewery syndrome) and probabilistic multi-omics associations, and strict equity and fairness monitoring to ensure evidence is not contributing to disparities. While ethical discussions of biology in criminal justice have long been shaped by cautionary narratives rooted in eugenics and related ideologies, advances in contemporary science—particularly recognition of widespread neurodiversity—now highlight ethical risks associated with *non-consideration* or *ignorance* of biological evidence.

Of particular concern is *strategic ignorance*: the deliberate avoidance of emergent evidence that challenges entrenched assumptions. For example, growing consensus views psychopathy as a spectrum-based neurodevelopmental condition, shaped by brain architecture and often compounded by trauma, rather than as an immutable moral failing (DeAngelis, 2022; Berluti et al., 2025; Pinto et al., 2025). Similarly, attention-deficit hyperactivity disorder—frequently overlapping with other neurodivergent conditions such as autism—is associated with elevated risks of substance use and justice involvement (Miller et al., 2025). Autistic traits have been reported to be an independent predictor of incarceration (Loureiro et al., 2018). Strategic ignorance of such findings enables continued moral condemnation and reinforces stigma toward individuals whose neurobiology constrains agency.

Emerging medical paradigms, including the proposed *Neurodiversity Framework in Medicine*, are reframing a broad range of neuropsychiatric conditions, including psychopathy, as expressions of neurodiversity rather than discrete pathologies (Miranda-Ojeda et al., 2025). This approach challenges diagnostic silos, reduces stigma, and aligns with personalized, precision-oriented care emphasized in public health–based justice models. Neurodivergent conditions are often examined in siloed ways, with less attention directed at the overlaps (Barnard-Brak and Richman, 2021). Increasingly, it is evident that traditional diagnostic categories fail to capture the continuous and heterogeneous nature of cognitive and neural variation, while individualized strategies can both respect neurodiversity and provide targeted support where needed (Mohammad et al., 2025).

These developments raise a broader ethical question: whether continued marginalization of biosciences within criminal justice paradigms is itself contributing to harm and stigmatization. This concern extends to education. It is ethically questionable that criminal justice degree programs—at undergraduate through doctoral levels—often lack mandatory training in biology, neuroscience, chemistry, or biopsychology, and are rarely supervised by faculty with bioscience expertise. Academic training shapes research priorities, policy development, and institutional norms. Viewed retrospectively, the science underpinning decades of mass incarceration and retributive policy was constructed almost entirely from social theory, largely excluding biology and ignoring neurodiversity (Delisi, 2013).

## 7 Conclusion—toward a biologically informed and ethically grounded justice system

This perspective has sought to unsettle the assumptions that continue to anchor criminal justice systems in prescientific views of agency and moral responsibility. The convergence of evidence from neuroscience, behavioral genetics, epigenetics, microbiome science, and the social determinants of health makes clear that justice-involved behavior cannot be understood—or addressed—through folk psychology alone. Ignoring this reality no longer reflects caution; it reflects epistemic inertia.

Legalomics provides a framework for integrating biological and environmental knowledge without collapsing into determinism or eroding public safety. By foregrounding neuroplasticity, biological constraint, and individual variability, it supports a justice paradigm oriented toward prevention, rehabilitation, and proportional restraint rather than moral desert. Within this frame, criminolytic interventions are not instruments of moral correction, but tools for supporting neurocognitive function and reducing harm under real-world constraints on agency.

As we argued above, education is a central component to challenging unscientific folk narratives and widening the lens of forensic neuroecology. To the extent that criminal justice degree programs remain dominated by social theories, exhibiting sustained resistance to biological perspectives on behavior, research investments will be minimal and the practical application of legalomics will remain stymied. The public health quarantine model deserves transdisciplinary attention and consideration for translation and tested implementation. The model supports rehabilitative programming but cautions against moralizing non-response. Overestimation of intervention efficacy—particularly when driven by strong agency assumptions—risks reproducing blame when individuals fail to improve.

The ethical challenge facing criminal justice is no longer whether biological science *could* be misused, but whether continued resistance to its careful, regulated inclusion perpetuates inequity, stigma, and avoidable harm. As understandings of neurodiversity, responsibility, and behavioral health continue to evolve, justice systems must adapt accordingly—developing frameworks that are scientifically grounded, ethically coherent, and responsive to human variation. Reimagining justice in this way does not diminish accountability. It redefines it—aligning legal practice with contemporary knowledge and orienting systems toward outcomes that better serve both societal protection and human dignity.
